# Effects of Near-Natural Forest Management on Soil Microbial Communities in the Temperate–Subtropical Transition Zone of China

**DOI:** 10.3390/microorganisms13081906

**Published:** 2025-08-15

**Authors:** Tian Zhang, Xibin Dong, Jin Yang, Zhenhua Li, Jiangxiong Zhu

**Affiliations:** 1 Department of Life Sciences, Yuncheng University, Yuncheng 044000, China; zhangtian@ycu.edu.cn (T.Z.); yangjin@ycu.edu.cn (J.Y.); lizhenhua@ycu.edu.cn (Z.L.); 2 Key Laboratory of Sustainable Forest Management and Environmental Microbial Engineering of Heilongjiang Province, Northeast Forestry University, Harbin 150040, China; 3 School of Agriculture and Biology, Shanghai Jiao Tong University, Shanghai 200240, China; zjx261023@163.com

**Keywords:** forest type, soil depth, soil microorganism, soil physicochemical properties, functional group

## Abstract

In order to precisely improve the quality of major tree species in northern China, near-natural differentiated management has been gradually introduced into forestry practice, aiming to optimize forest structure, enhance forest quality, and promote nutrient cycling and water conservation. As an essential element of forest ecosystems, soil microbes contribute to biodiversity preservation and nutrient turnover in soils. This study selected three typical forest types (*Quercus acutissima* forest, *Pinus tabulaeformis* forest, and *Pinus tabulaeformis* × *Quercus* mixed forest) that have been managed with target trees on Zhongtiao Mountain. Using 16S/ITS rRNA high-throughput sequencing, this study systematically assessed the influences of forest type and soil depth (0–60 cm) on the soil properties and microbial communities. The results showed that the fungal alpha diversity indices were the highest in *Pinus tabulaeformis* forest, which decreased with soil depth. Actinobacteriota exhibited the greatest relative abundance in mixed forest, whereas Ascomycota predominated in the *Pinus tabulaeformis* forest. The microbial co-occurrence network exhibited greater complexity compared to the pure forest. Microbial carbon and nitrogen cycling functions showed strong correlation with soil pH and nutrient levels. Symbiotrophs dominated the fungal community, and ectomycorrhizae were significantly abundant in mixed forests. pH is the dominant factor driving changes in microbial communities. In summary, the mixed forest improved soil nutrients, enhanced the complexity of microbial networks, and supported higher ectomycorrhizal abundance. These findings provide practical guidance for improving soil health and stability of forest ecosystems through near-natural management.

## 1. Introduction

Recognized as key drivers of terrestrial biodiversity [1,2], soil microbes play indispensable roles in mediating biogeochemical cycles [3]. Microbial diversity and functional attributes in forest soils play a vital role in driving key ecological processes, including organic matter degradation, nutrient turnover, vegetation productivity, and carbon flux dynamics [4,5,6]. Microbial assemblages in soil are primarily composed of bacteria and fungi, which significantly contribute to nutrient mineralization and soil health [7,8]. Bacteria participate in carbon, nitrogen, and phosphorus cycling and modulate mycorrhizal interactions [9]. On the other hand, fungi are essential for litter decomposition and soil structure formation. Their responses to vegetation and environmental conditions differ markedly [10,11,12,13]. For example, bacteria tend to converge under low pH, whereas fungi show higher beta diversity in acidic soils [14]. Fungi also exert stronger influence on decomposition regardless of litter type [15] and may benefit more from species richness [16]. Additionally, bacterial network complexity decreases from cold to tropical zones, while fungal networks show the opposite trend [17]. Therefore, understanding microbial community structure, particularly bacterial–fungal interactions, is essential for revealing soil ecological functions.

Soil microorganisms, plant roots, and the mineral matrix form an interdependent network that regulates ecosystem functions [18]. Tree species shape heterogeneous microhabitats through root exudates, litter quality, and secondary metabolites [19,20,21,22], thereby influencing soil microbial communities and altering nutrient transformation and soil nutrient cycling [23]. Shifts in forest types and species composition lead to changes in microbial diversity and function [24], especially between coniferous and broadleaf species [25], which differ in litter chemistry and decomposition strategies. The litter of coniferous tree species is rich in high-C/N-ratio substances such as tannins and lignin, which can enrich fungi with the ability to degrade lignocellulose [26], while broadleaved trees are more conducive to the colonization of eutrophic bacteria by releasing soluble sugars and organic acids. At the same time, the decomposition rate of forest litter and the release rate of plant nutrients in coniferous species were lower [15], showing strong acidification ability [27]. However, earlier studies have also shown that the influence of plants on bacterial communities may not be evident in many ecosystems [11,28]. Factors such as climate, parent material, and management practices may also shape microbial communities [23,29,30]. Although previous studies have revealed close plant–microbe interactions and the underlying regulatory processes of microbial communities, their specific responses across climatic gradients and soil conditions remain poorly understood. Therefore, investigating microbial assemblages across forest types is essential to enhance our understanding of ecosystem nutrient cycling and stability.

The richness and heterogeneity of plant species are closely associated with shifts in soil microbial communities [31,32]. Most studies suggest that mixed forests support higher microbial diversity than pure forests due to richer root exudates and organic matter inputs, which create varied ecological niches [33]. However, elevated tree diversity does not necessarily enhance microbial alpha diversity, and in some cases, higher plant diversity may even reduce microbial diversity, likely due to variations in scale, site conditions, or assessment methods [2,34]. Forest soils also show strong vertical stratification: surface soils, enriched by litter inputs, exhibit higher microbial activity and nutrient content, while deeper layers receive less organic matter and show lower microbial biomass and enzyme activity [33,35]. Although microbial abundance often declines with depth [36], deep-soil microorganisms still contribute to nutrient cycling and soil formation [37,38]. Thus, examining the soil characteristics that vary with depth is essential for understanding belowground ecological processes.

Zhongtiao Mountain is situated in southern Shanxi Province, China, nestled between the Taihang and Huashan mountain ranges. It lies within a climatic ecotone that spans both temperate and subtropical zones and hosts the richest tree species diversity in Shanxi. Due to sharp environmental gradients and ecosystem instability, these zones are highly sensitive to climate change [39,40]. Currently, research predominantly concentrates on terrestrial ecosystems within well-defined climate zones [11,41], with the dynamics of soil microorganisms in transitional climatic areas remaining poorly understood. For example, it was reported that the microbial OTU compositions and functional gene network of the Funiu Mountain (temperate–subtropical climate transition zone) differed significantly (*p* < 0.005) from those of Wulu Mountain (temperate climate) and Shennongjia Mountain (subtropical climate), with low redundancy in microbial function along climatic transition zones [42]. Therefore, investigating variations in soil properties in such regions is essential for understanding vegetation–microbe interactions, and provides a theoretical basis for understanding the adaptive mechanisms of forest ecosystems in response to climate change.

According to the actual situation of land conditions, forest types, development stages, dominant functions, and management objectives, Qijiahe Forest Farm on Zhongtiao Mountain has established a near-natural differentiated management technology system. This system incorporates various management modes and stand types, aiming to simulate natural forest development with minimal intervention and to promote ecological stability and biodiversity through the establishment of mixed and native-dominant forests with reduced silvicultural disturbance. In this study, we selected three forest types (*Quercus acutissima* (QA), *Pinus tabulaeformis* (PT), and coniferous and broadleaved mixed forest (*Pinus tabulaeformis* × *Quercus*) (MF)), investigating the soil microbial diversity, composition, co-occurrence network, and functional prediction in different forest types at different soil depths. Specifically, we therefore formulate the following hypotheses: (1) Higher levels of microbial diversity are predicted to occur in mixed forest. (2) Bacterial and fungal communities are both markedly influenced by forest type, but changes in soil depth predominantly influence fungal communities. (3) Soil physiochemical properties strongly influence the microbial community and function.

## 2. Materials and Methods

### 2.1. Study Area and Soil Sampling

This study was conducted at the Qijiahe Forest Farm, located in the Zhongtiao Mountain area of Shanxi Province, China (34°58′~35°10′ N, 111°27′~111°40′ E), with an elevation range of 350~1 566 m. This region experiences a warm temperate climate under the influence of the continental monsoon. Due to the influence of mountainous terrain, it locally exhibits characteristics of a transition zone from semi-humid to semi-arid climate. The mean annual temperature is approximately 12.9 °C, with recorded extremes reaching 40.2 °C in summer and dropping to −18.3 °C in winter. Annual precipitation ranges between 500 and 720 mm, predominantly occurring in July and August. The region receives around 2400 h of sunshine annually, and the frost-free season extends for about 205 days. The terrain within the area consists of undulating erosional low-to-mid mountains, with rocks mainly composed of Archean phyllite limestone, covered on the surface by Quaternary loess parent material. The main tree species are *Quercus acutissima*, *Quercus variabilis*, *Quercus liaotungensis*, and *Pinus tabuliformis.* The zonal soil is brown soil, with a forest canopy closure of 0.8 and a northwest-facing slope.

In July 2024, within the experimental forest area, three forest types were selected: *Quercus acutissima* forest (QA), *Pinus tabuliformis* forest (PT), and a coniferous–broadleaf mixed forest (*Pinus tabulaeformis* × *Quercus*) (MF). For each forest type, three 20 m × 20 m plots were randomly established, with spacing greater than 10 m. In each plot, three subplots were arranged diagonally to capture spatial variability. GPS was used to record the geographical coordinates of each plot, along with basic site conditions including elevation, slope aspect, slope position, canopy closure, average diameter at breast height (DBH), average tree height, and stand density (Table 1).

In each subplot, the top layer of withered material was first removed, and then according to the soil occurrence level [43], samples were taken from three depth intervals—0–10 cm, 10–20 cm, and 20–40 cm—using a soil auger with a 5 cm diameter. For each plot, soil cores from the three subplots were pooled by depth to form composite samples. In total, 27 samples were obtained from the nine plots and three depth layers. Each composite sample was subsequently divided into two aliquots, with one portion transferred into sterile tubes and stored at −80 °C, for high-throughput sequencing, while the other was kept in sealed bags, air-dried, and sieved through a 2 mm mesh after removing visible roots, gravel, and organic debris, to prepare for physicochemical property analysis.

### 2.2. Analysis of Soil Physical and Chemical Properties

Soil physical properties were analyzed by the ring knife technique and indices including soil bulk density (BD) and total porosity (PO). Soil pH was assessed by the potentiometric approach [44]. Total nitrogen (TN) was quantified through Kjeldahl digestion. Mo-Sb colorimetric analysis of NaOH alkali dissolution was used to acquire total phosphorus (TP) [45]. Sodium hydroxide fusion combined with the flame photometric method was used to evaluate total potassium (TK). The same flame photometric method was applied to quantify the available potassium (AK) which was extracted with ammonium acetate [46]. Organic matter (SOM) and organic carbon (SOC) contents were determined through the methodology by the oxidation method with potassium dichromate [47].

### 2.3. Soil DNA Extraction, PCR Amplification, and High-Throughput Sequencing

Genomic DNA was extracted from soil samples using the TIANamp kit (DP304; Tiangen Biotech, Beijing, China). Extracted DNA was examined by 1% agarose gel electrophoresis, and its concentration and purity were quantified with a NanoDrop 2000 spectrophotometer (Thermo Fisher Scientific, Waltham, MA, USA). For bacterial community profiling, the V3–V4 region of the 16S rRNA gene was amplified with primers 341F (5′-CCTAYGGGRBGCASCAG-3′) and 806R (5′-GGACTACNNGGGTATCTAAT-3′). The ITS1 region of fungi was amplified using primers ITS5-1737F and ITS2-2043R. PCR amplicons were confirmed on 2% agarose gels and subsequently purified using the Universal DNA Purification Kit (DP214; Tiangen Biotech, Beijing, China). Sequencing libraries were prepared using the NEBNext^®^ Ultra™ II DNA Library Prep Kit (E7645B; New England Biolabs, Ipswich, MA, USA), followed by sequencing on the Illumina NovaSeq 6000 platform (Illumina, San Diego, CA, USA) at Novogene (Beijing, China). The resulting sequencing datasets have been submitted to the NCBI Sequence Read Archive (SRA) under accession numbers PRJNA1267701 (bacterial) and PRJNA1267702 (fungal).

### 2.4. Sequence Analysis

The initial sequencing reads were combined utilizing FLASH software (version 1.2.11) and subsequently processed with Cutadapt to eliminate adapter and primer sequences [48]. Quality control was carried out using Fastp (v0.23.1) [49], yielding 1,469,032 high-quality bacterial 16S rRNA sequences (55,801–116,135 per sample) and 1,484,629 fungal ITS sequences (51,786–116,904 per sample). Denoising, chimera removal, and error correction were conducted using DADA2 in QIIME2 to generate amplicon sequence variants (ASVs) [50]. This process yielded 32,614 bacterial ASVs and 7896 fungal ASVs. Representative ASV sequences were taxonomically assigned using the Silva v138.1 (bacteria) and UNITE v9.0 (fungi) databases [51].

### 2.5. Statistical Analysis

R software (version 4.4.3) was employed to perform all statistical analyses of the data. Analysis of variance (ANOVA) was performed to evaluate the influence of forest type and soil depth, and multiple comparisons were carried out using the Tukey HSD test. β-diversity was examined using principal coordinate analysis (PCoA) based on weighted UniFrac distances (phyloseq and vegan packages), and community compositional variation was tested through PERMANOVA (adonis function). Redundancy analysis (RDA) with Monte Carlo permutations explored microbial–environment relationships. Spearman’s correlation was employed to link microbial composition with environmental factors. Microbial co-occurrence networks were built using ASVs with >0.05% relative abundance and significant correlations (|r| > 0.8, *p* < 0.01, Spearman). Network construction was conducted using the igraph package [52], and visualization was achieved via Gephi (v0.10.1, Fruchterman–Reingold layout). Key topological features included node and edge counts, proportions of positive/negative correlations, average degree, density, mean path length, clustering coefficient, and modularity. The microbial network complexity index was then calculated as the unweighted mean of the Z-scores for the number of nodes and edges, average degree, average clustering coefficient, network density, and the inverse of the average path length [53]. Functional prediction of bacterial and fungal communities was performed using FAPROTAX and FUNGuild, respectively.

## 3. Results

### 3.1. Analysis of Soil Physicochemical Properties

**Figure 1:**
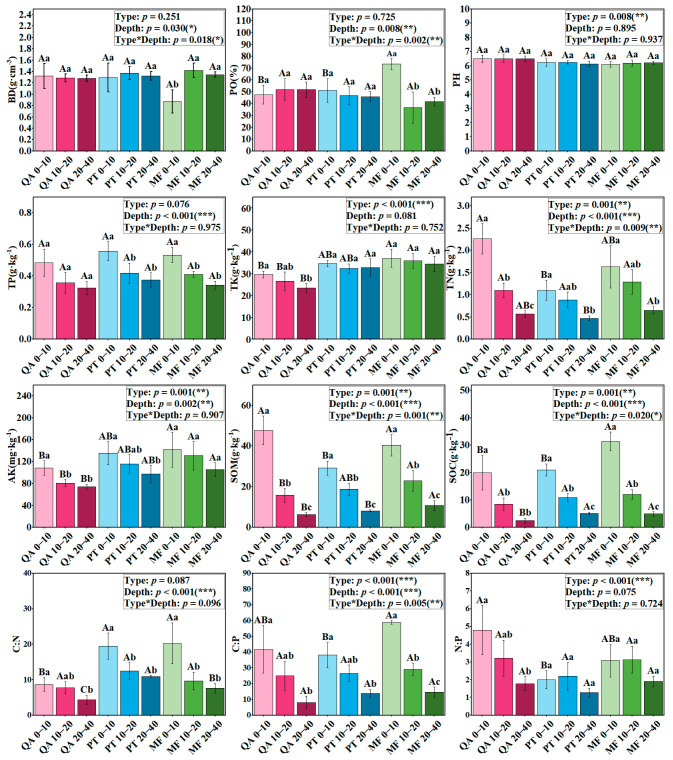
Variation in soil properties across different forest types and soil depths. This figure shows the significance (*p* values) associated with stand type, soil profile depth, and the combined influence of both on each measured soil attribute: ***—*p* < 0.001; **—*p* < 0.01; *—*p* < 0.05. Lowercase letters indicate significant differences among soil layers within the same forest type, while uppercase letters denote differences among forest types at the same soil depth, based on Duncan’s test (*p* < 0.05). Note: QA, *Quercus acutissima*; PT, *Pinus tabuliformis*; MF, *coniferous and broadleaved mixed forest*; 0–20, soil depth (0–20 cm); 20–40, soil depth (20–40 cm); 40–60, soil depth (40–60 cm); TP, total phosphorus; TK, total potassium; TN, total nitrogen; AK, available potassium; SOM, soil organic matter; SOC, soil organic carbon.

Significant variation in soil properties was observed across different forest types and soil depths (Figure 1). Both bulk density (BD) and total porosity (PO) were notably influenced by soil depth (*p* < 0.05; Table S1). BD exhibited an initial rise followed by a decline as soil depth increased, with the minimum values observed in the topsoil, while PO showed the opposite pattern. At the 0–10 cm depth, PO in MF was markedly greater than that observed in QA and PT (*p* < 0.05). In the deeper layers, although QA exhibited the highest value and the lowest was demonstrated in MF, the differences in PO content were not significant.

Forest types had significant effects on soil pH, TK, and N:P (*p* < 0.05) (Table S1). The TK content of MF (34.45~37.03 g·kg^−1^) was significantly higher than that of QA (23.43~29.59 g·kg^−1^) (*p* < 0.001). QA exhibited a significantly elevated N:P ratio compared to the other two forest types, particularly evident within the top 0–10 cm of the soil profile (*p* < 0.001). TN, AK, SOM, SOC, and C:P were all strongly influenced by forest type and depth (Table S1). The contents of AK (105.28~141.85 mg·kg^−1^) and SOC (4.94~31.36 g·kg^−1^) of MF were markedly greater compared to QA (74.07~108.11 mg·kg^−1^, 2.05~19.91 g·kg^−1^). Within the topsoil layer (0–10 cm), MF exhibited a significantly higher C:P ratio than PT (*p* < 0.05), whereas QA recorded significantly elevated TN and SOM values relative to PT (*p* < 0.05). At depths of 10–40 cm, SOM levels in MF exceeded those of the other forest types (*p* < 0.05).

### 3.2. Alpha Diversity of Soil Microbial Communities

**Figure 2:**
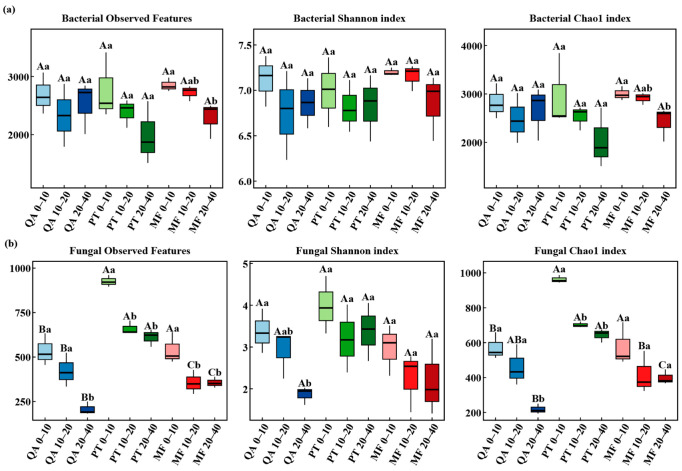
α-diversity (including observed features, Shannon and Chao1 indices) of bacterial (**a**) and fungal (**b**) communities. Lowercase letters denote significant differences among soil depths within the same forest type, while uppercase letters represent differences among forest types at the same soil depth, based on Duncan’s test (*p* < 0.05). Note: QA, *Quercus acutissima*; PT, *Pinus tabuliformis*; MF, *coniferous and broadleaved mixed forest*; 0–20, soil depth (0–20 cm); 20–40, soil depth (20–40 cm); 40–60, soil depth (40–60 cm).

Alpha diversity is used to assess the diversity level of soil microbial communities, reflecting both species richness and evenness [54]. According to two-way ANOVA (Table S2), the observed bacterial species richness was notably impacted by soil depth (*p* < 0.05). In particular, bacterial richness and diversity in MF soils decreased with depth, and surface soils exhibited significantly greater bacterial diversity indices than deeper layers (*p* < 0.05) (Figure 2). In contrast, Shannon and Chao1 indices did not differ significantly among forest types or across soil depths (*p* > 0.05). In contrast, fungal community diversity was more significantly influenced by forest types and soil depths (*p* < 0.05) (Table S2). For example, PT had significantly higher fungal richness and Chao1 values compared to QA and MF (*p* < 0.05), particularly in the 0–10 cm layer, which exceeded values observed at a depth of 20–40 cm (*p* < 0.05; Figure 2).

### 3.3. Beta Diversity of Soil Microbial Communities

**Figure 3:**
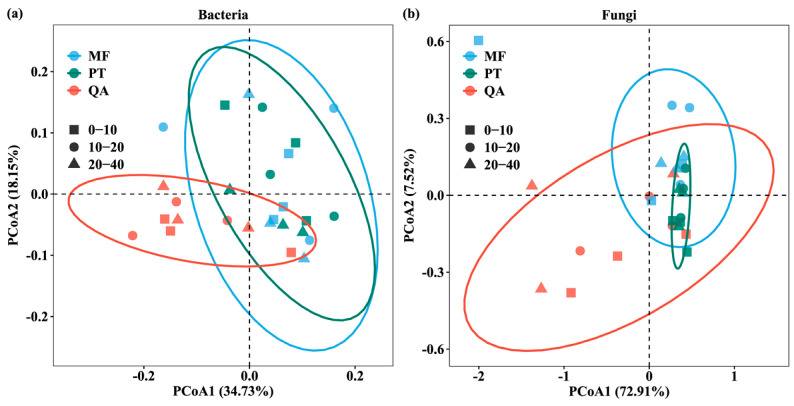
Principal coordinate analysis (PCoA) of soil bacterial (**a**) and fungal (**b**) communities based on weighted UniFrac distance analysis of ASV levels. Colors represent forest types and shapes represent soil depths. Each ellipse represents the 95% confidence interval of samples from the same forest type. PCoA1 and PCoA2 explain the variation in community composition among samples.

Beta diversity reflects compositional dissimilarity or similarity in soil microbial communities [54]. Principal coordinate analysis (PCoA) was applied to assess structural variations in bacterial and fungal communities across the three forest types (Figure 3). The first two principal coordinates (PC1 and PC2) accounted for 52.88% and 80.43% of the total variation in bacterial and fungal communities, respectively. PERMANOVA results also showed that forest types had a significant effect on the structure of bacterial and fungal communities (*p* < 0.05) (Table 2). Neither soil depth nor its interaction with forest type significantly influenced the microbial community structure (*p* > 0.05).

### 3.4. Composition of Soil Bacteria and Fungi

**Figure 4:**
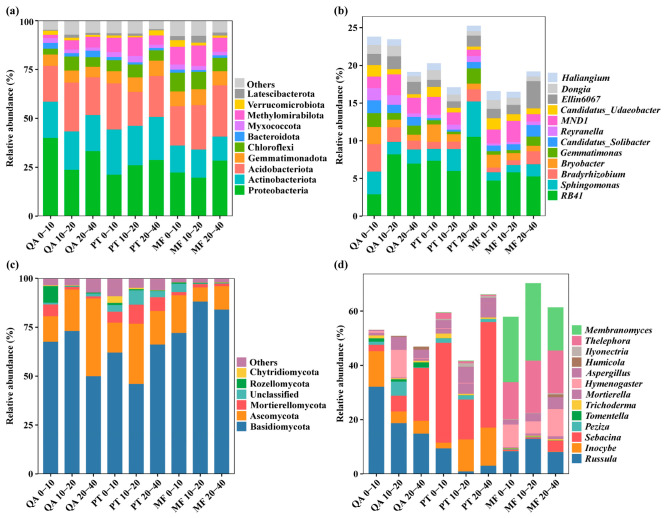
Relative abundance of microbial community composition in three forest types. (**a**) Phylum-level bacterial taxa; (**b**) genus-level bacterial taxa; (**c**) phylum-level fungal taxa; (**d**) genus-level fungal taxa. Only taxa with relative abundance >1% are shown; all other taxa are grouped into “Others”.

Analysis at the phylum level revealed ten bacterial phyla and twelve genera exhibiting relative abundances over 1%, among which the top three soil bacterial phyla were Proteobacteria (19.71~40.07%), Acidobacteriota (17.37~26.21%), and Actinobacteriota (12.24~23.06%) (Figure 4a), and the bacterial genera were *RB41* (2.90~10.53%), *Sphingomonas* (0.97~4.69%), and *MND1* (0.84~2.79%) (Figure 4b). Forest type significantly influenced the relative abundance of certain bacterial phyla (Actinobacteriota, Chloroflexi, Bacteroidota, Methylomirabilota, and Latescibacterota) and bacterial genera (*Sphingomonas*, *Haliangium*, *Candidatus_Solibacter*, *Bryobacter*, *Dongia*, and *Ellin6067*) (*p* < 0.05) (Table S3), among which Actinobacteriota, Chloroflexi, Methylomirabilota, and Latescibacterota were most abundant in MF, while QA exhibited the highest proportion of Bacteroidota. The composition of bacterial phyla and genera remained largely unaffected by soil depth, with the exception of *RB*41. The relative abundance of *RB41* was significantly greater in deeper soil layers compared to surface soils (*p* < 0.05).

Among fungi, five phyla with relative abundances exceeding 1% were identified. The predominant fungal groups across all plots were Basidiomycota (46.04–88.15%) and Ascomycota (7.27–39.74%) (Figure 4c). According to two-way ANOVA, the abundance of fungal phyla (Ascomycota, Mortierellomycota) and genera (Mortierella, Sebacina, Ilyonectria, Membranomyces) varied significantly across forest types (*p* < 0.05; Table S4). Ascomycota showed the highest proportion in PT soils at 0–20 cm, while Mortierellomycota peaked at 20–40 cm. At the genus level, Sebacina and Ilyonectria were most enriched in PT, whereas Membranomyces was dominant in MF soils.

### 3.5. Soil Microbial Community Co-Occurrence Network Analysis

**Figure 5:**
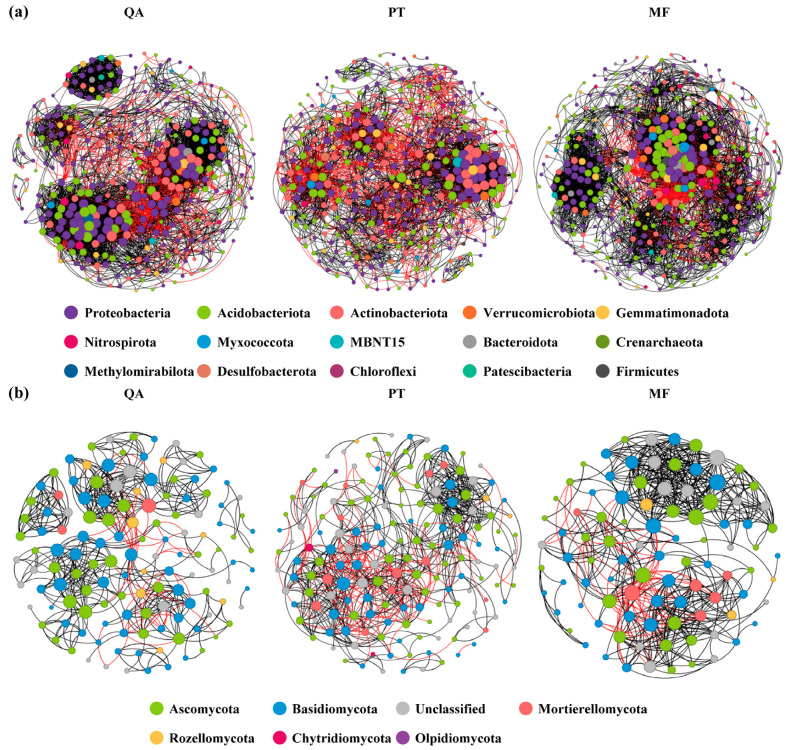
Microbial co-occurrence networks constructed at the phylum scale across three forest types: bacteria (**a**) and fungi (**b**). Black lines denote significant positive associations, while red lines represent significant negative ones. Each node corresponds to an ASV, and its size reflects the degree value, i.e., the total number of connections.

Co-occurrence network analysis revealed that MF exhibited the most intricate bacterial and fungal interactions, as indicated by the network complexity index (Figure 5, Table 3). The bacterial network in MF contained 407 nodes and 5745 edges, with a higher density and average degree, and a greater proportion of positive associations, resulting in a structurally more intricate network. QA and PT networks had fewer nodes and edges, with complexity indices of 0.034 and −0.922, respectively (Table 2). For fungi, MF had the highest edge number (520) and average degree (11.183), despite having fewer nodes (93), forming a highly clustered structure. QA and PT had more nodes (130 and 166) but fewer edges (477 and 695) and lower average degrees (7.338 and 8.373), with the lowest complexity in QA (−0.342) and intermediate complexity in PT (−0.089) (Table 2). Overall, bacterial networks exhibited more nodes and edges than fungal ones across all forest types, indicating greater structural complexity and interaction frequency, while fungal networks were more modular and sparse.

### 3.6. Functional Prediction of Soil Microbial Communities

**Figure 6:**
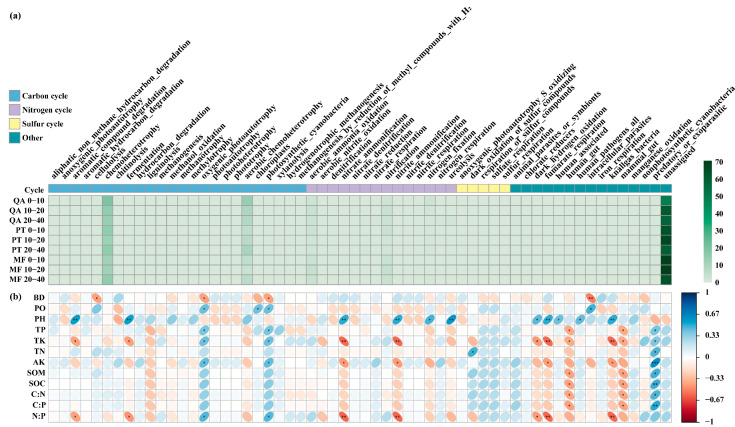
Prediction and environmental correlation of soil bacterial functions. (**a**) Heat map of predicted bacterial functional profiles. (**b**) Associations between bacterial functions and soil parameters based on Spearman correlation. *: *p* < 0.05, **: *p* < 0.01, ***: *p* < 0.001.

According to FAPROTAX functional annotation, 57 bacterial functional groups were classified (Figure 6a), including 24 related to the carbon cycle, 14 to the nitrogen cycle, and 5 to the sulfur cycle. The most abundant functions (relative abundance > 1%) included chemoheterotrophy (12.45~19.49%), aerobic_chemoheterotrophy (4.21~14.44%), aerobic_ammonia_oxidation (1.98~4.25%), nitrification (1.98~4.25%), nitrogen_fixation (0.58~2.44%), ureolysis (0.12~2.07%), predatory_or_exoparasitic (0.49~1.07%), and nitrate_reduction (0.31~1.22%). The relative abundance of aerobic_chemoheterotrophy differed significantly across forest types (*p* < 0.001; Table S5), with QA exhibiting the highest functional values. Correlation analysis based on Spearman’s method (Figure 6b) revealed that soil pH was positively associated with the majority of bacterial functions (*p* < 0.05). Within the carbon cycle, oxygenic_photoautotrophy and photosynthetic_cyanobacteria were positively associated with TK, TP, AK, and the N:P ratio (*p* < 0.05). Within the nitrogen cycle, both nitrate_ammonification and nitrite_ammonification were inversely associated with TK and the N:P ratio (*p* < 0.05). Additionally, hydrogen metabolism-related functions such as dark_hydrogen_oxidation and knallgas_bacteria were also negatively associated with soil nutrients (*p* < 0.05). Notably, the predatory_or_exoparasitic function showed significant positive correlations with most soil nutrient indicators (*p* < 0.05).

**Figure 7:**
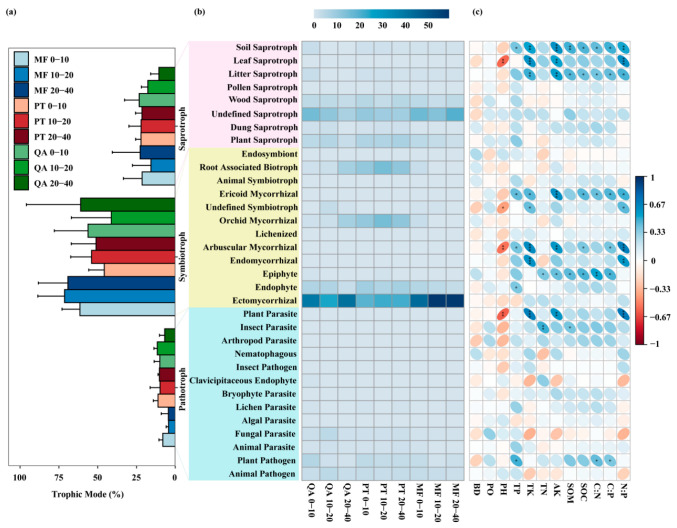
Variations in soil fungal communities based on major trophic strategies and guild classifications (FUNGuild). (**a**) Distribution of fungi by trophic mode; (**b**) dominant fungal guilds; (**c**) Spearman correlation between fungal functions and soil variables. *: *p* < 0.05, **: *p* < 0.01, ***: *p* < 0.001.

FUNGuild analysis revealed that symbiotrophs dominated the fungal community across all forest types, accounting for 41.25~71.37%, followed by saprotrophs (10.56~23.27%) and pathotrophs (4.35~11.60%) (Figure 7a). The dominant symbiotrophic groups included ectomycorrhizal, orchid mycorrhizal, root-associated biotroph, and endophyte (Figure 7b), with the first three significantly influenced by forest types (*p* < 0.05) (Table S6). Ectomycorrhizal abundance was highest in MF, while orchid mycorrhizal and root-associated biotroph were more abundant in PT than in QA and MF. Saprotrophs were predominantly composed of plant- and wood-associated groups (Figure 7b), with plant saprotrophs significantly more enriched in PT compared to MF (*p* < 0.05; Table S6). Pathotrophs mainly consisted of animal pathogens, fungal parasites, and plant pathogens (Figure 7b), but their abundance was not significantly influenced by forest type or soil depth (*p* > 0.05; Table S6). Symbiotic and saprophytic trophic modes were positively correlated with soil physicochemical factors (Figure 7c).

### 3.7. Correlations Between Soil Parameters and Soil Microbial Communities

**Figure 8:**
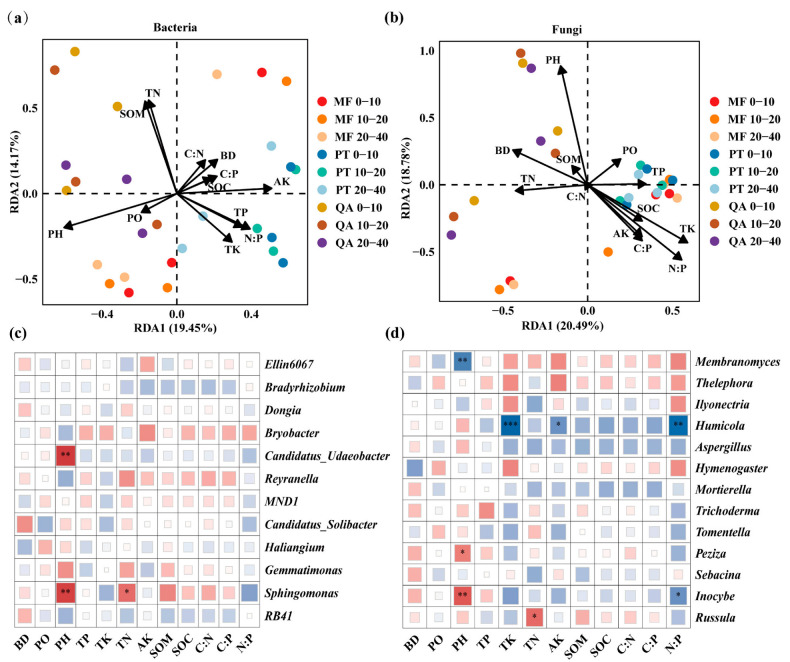
RDA based on the level of ASVs: (**a**) bacteria and soil properties, (**b**) fungi and soil properties, (**c**) heat map of Spearman’s correlation coefficients between bacterial genera with relative abundance >1% and soil properties, and (**d**) heat map of Spearman’s correlation coefficients between fungal genera with relative abundance >1% and soil properties. Note: Blue color indicates negative correlation, red color indicates positive correlation, and darker color indicates higher correlation. *: *p* < 0.05, **: *p* < 0.01, ***: *p* < 0.001.

To explore the relationships linking environmental factors and microbial communities, RDA and Spearman’s rank correlation analysis were employed. RDA results indicated that soil physicochemical conditions accounted for 33.62% of the variation in bacterial community composition (Figure 8a), with pH, TN, and AK identified as the primary contributing factors (Table S7). Spearman correlations revealed strong positive associations between pH and *Sphingomonas* and *Candidatus_Udaeobacter* (*p* < 0.01), and between TN and *Sphingomonas* (*p* < 0.05) (Figure 8c). For fungi, RDA axes explained 39.27% of community variation (Figure 8b), slightly more than for bacteria. Key drivers included pH, N:P, TK, and AK (Table S5). A significant positive association was identified between pH and Inocybe (*p* < 0.01), as well as Peziza (*p* < 0.05), while a negative correlation was observed with *Membranomyces* (*p* < 0.01). *Humicola* abundance was negatively associated with TK (*p* < 0.001), N:P (*p* < 0.01), and AK (*p* < 0.05) (Figure 8d).

## 4. Discussion

### 4.1. Variation in Soil Properties Across Forest Types and Soil Depths

This study revealed a notable decline in soil nutrients with increasing depth, aligning with findings from most previous research [55,56], with this pattern being attributed to greater litter input and biological residues in surface soils, as well as fine root distribution. Earlier studies have shown that over 70% of live roots and 60% of dead fine roots are mainly distributed within the 0–20 cm layer of soil in temperate forests [57]. Our results further revealed that the concentrations of TK, AK, and SOC were markedly higher in mixed forests than in pure forest stands (Figure 1). This could be attributed to higher litter accumulation observed in mixed forests (9.66 t·hm^−2^), versus *Pinus tabulaeformis* (7.63 t·hm^−2^) and *Quercus acutissima* (4.67 t·hm^−2^), as litter input constitutes a key origin of soil organic matter. Mixed forests have been reported to possess higher capacities for nutrient retention [58] and enhance litter decomposition and TOC accumulation [59,60,61]. The *Quercus acutissima* forest at the research site is currently in the fast-growing period (Table 3) [62], which increases nutrient uptake and may contribute to nutrient depletion in pure stands. Potassium, being highly mobile, is more easily leached in broadleaf forests [63]. However, TN and SOM in the 0–10 cm layer of *Quercus acutissima* forest were higher than those of the *Pinus tabulaeformis* forest (Figure 1), accompanied by a much higher N:P ratio (4.80 vs. 2.00) and a lower C:N ratio (8.67 vs. 19.43). This indicates that nitrogen is abundant in the *Quercus acutissima* forest, which enhances microbial utilization of organic matter and accelerates nutrient mineralization [64], while the higher N:P ratio suggests that microbial processes in this forest may be potentially limited by phosphorus, consistent with the common pattern of microbial phosphorus limitation in forest soils [65].

### 4.2. Variation in Soil Microbial Community Across Forest Types and Soil Depths

This study showed that soil depths significantly affected soil microbial diversity. Specifically, the fungal diversity index of the surface soil exhibited a markedly higher value than in deeper soil layers (Figure 2a). This indicates that the surface soil creates a more favorable environment, characterized by increased organic input and improved oxygen availability [28]. There were no significant differences in the Chao1 and Shannon indices of bacteria between different forest types (Table S2); some studies have shown that microbial diversity may remain stable despite differences in soil properties induced by forest type [66]. Fungal alpha diversity was markedly affected by forest type and soil depth (Table S2), suggesting a complex response of fungi to soil conditions across forest types [67]. *Pinus tabulaeformis* forest showed the highest fungal diversity (*p* < 0.05) (Figure 2b), supporting the idea that pure forest may enhance fungal richness [33]. Factors such as litter quality, pH, root systems, and microhabitats shape microbial composition and distribution [68]. Needle litter decomposes slowly and contains abundant structural carbon compounds, including lignin and resin, forming a stable carbon input and providing an ecological niche for different fungal groups [69]. In contrast, rapid litter decomposition in *Quercus acutissima* may intensify resource competition and suppress fungi. Additionally, lower pH in the soil of *Pinus tabulaeformis* may limit bacterial dominance, indirectly benefiting fungi. We also found that the fungal diversity in mixed forests (ASVS and Chao1) was lower than that in *Pinus tabulaeformis forests*, or showed no significant difference in the Shannon index. This pattern may be attributed to a higher degree of niche overlap in mixed forests, which leads to more complex microbial interactions and intensified interspecific competition. Under such competitive conditions, certain functional dominant groups may prevail, suppressing the survival of other species and ultimately reducing overall species richness [70]. The PCoA revealed that forest types significantly affected the beta diversity of microbial communities (*p* < 0.01) (Figure 3, Table 2), which might be associated with the distinct sensitivities of microbial communities to soil attributes under contrasting forest types.

Shifts in plant composition can indirectly shape soil microbial structures by altering both the biochemical profile of litter and the release of root-associated metabolites. Certain specific microbial communities respond differently to heterogeneous habitats, reflecting microbial niche differentiation [71,72]. Numerous investigations have reported that the dominant bacterial communities in soil are basically similar in different ecosystems. For example, Proteobacteria, Acidobacteriota, and Actinobacteriota dominate in forest soils [39], farmland soils [73], and alpine meadow soils [74], which aligns well with findings of the present research (Figure 4a). The relative average abundance of Actinobacteriota in the soil of mixed forests was significantly the highest (*p* < 0.05) (Table S3); this phylum is known to enhance key carbon pools and nitrogen availability, while reducing the C:N ratio [75]. The individual proportions of Proteobacteria and Actinobacteriota each exceeded 20.5% within the microbial consortium, reflecting conditions favorable for the survival of eutrophic and acidophilic bacteria [76]. Forest type did not significantly affect the two bacterial phyla (Table S3), indicating that the microbial community in a certain area is more influenced by edaphic conditions, and the dominant bacterial taxa serve crucial functions in structuring forest soil systems and regulating nutrient dynamics [66,77]. In this study, soil depth showed no evident impact on the composition of bacteria at the phylum and genus levels (except *RB41*) (Table S3). Evidence from earlier studies suggests that depth-related changes in bacterial communities are often site-specific and depend on soil profile characteristics, which may explain the lack of significant variation [78]. Notably, significantly higher levels of *RB41* were observed in deeper soils than in surface counterparts (*p* < 0.05). *RB41* is known to be an oligotrophic bacterial group that is negatively correlated with soil organic carbon concentration [79,80], and its enrichment in deeper layers suggests its potential role in the turnover of stable carbon pools in subsoils.

In this study, we found that Basidiomycota (46.04–88.15%) and Ascomycota (7.27–39.74%) were identified as the predominant fungal taxa (Figure 4c), with both participating in transformation of recalcitrant organic matter [81,82]. Ascomycota and Mortierellomycota were found to be significantly affected by forest types (Table S4), with Ascomycota showing a significant enrichment in the topsoil (0–20 cm) of *Pinus tabulaeformis* forest. Ascomycota is a common group of fungal communities in coniferous forests [83]. Basidiomycota has been identified as favoring habitats with high fertility, and may gradually be replaced by oligotrophic Ascomycota as nutrient status decreases [84].

### 4.3. Effect of Forest Types on Microbial Co-Occurrence Network

The microbial networks associated with mixed forests demonstrated higher complexity compared to those found in pure forests (Figure 5), as indicated by metrics such as node count, edge quantity, mean degree, network density, and minimal path length. This aligns with findings that network complexity increases with soil nutrient levels [85], and may also be enhanced by higher tree diversity, litter input, and root exudates [86]. Complex networks improve efficiency in the transfer of resources and information, plant growth and stress resistance, and ecological stability [87]. This suggests that maintaining high tree species diversity can support more complex and stable microbial networks.

Furthermore, we found that the microbial network in the mixed forest exhibited the shortest average path length and the lowest modularity among all forest types (Table 3), while maintaining a relatively high clustering coefficient. This suggests a small-world network structure featuring strong clustering tendencies and reduced mean path distances, which has been linked to enhanced resource utilization efficiency and improved network stability [88]. Previous studies have pointed out that if environmental filtering dominates and selects for microbial groups with similar functions or traits, the network modularity may decrease; however, if diverse resources promote functional differentiation and niche partitioning among microbes, the modularity may increase [89].

### 4.4. Variations in the Functions of Soil Microbial Communities Across Forest Types

Microorganisms play key roles in nitrogen, sulfur, and carbon cycles [90], and in our study, 24 functions related to the carbon cycle, 14 to the nitrogen cycle, and 5 to the sulfur cycle were detected (Figure 6a). There were synergistic or antagonistic relationships between different microbial functions and soil nutrients [91]. For example, photosynthetic microorganisms (oxygenic_photoautotrophy, photosynthetic_cyanobacteria) were positively correlated with most soil indicators; in the nitrogen cycle, nitrate_ammonification and nitrite_ammonification showed significant negative correlations with total potassium (TK) and the N:P ratio (*p* < 0.05) [92]. Hydrogen metabolic function is more important in oligotrophic systems as it is usually a complementary metabolic pathway, and the advantage is manifested in resource-poor or anaerobic conditions [93].

FUNGuild annotations indicated that ectomycorrhizal fungi belonging to the symbiotic trophic functional group were the main trophic modes (Figure 7a,b), and this group of fungi occupies a core functional position in forest ecosystems [94]. Ectomycorrhizal fungi were most abundant in mixed forests (Table S6), possibly reflecting the presence of a more complex or diversified plant inter-root system [95], which may offer increased ecological niches and carbon sources [96]. In this study, a significantly higher abundance of plant saprotrophs was detected in the *Pinus tabulaeformis* forest than in the mixed forest (Table S6), where enhanced root biomass and SOC after mixing may reduce carbon limitation, suppressing saprotrophic fungi [97,98], and previous findings suggested that higher levels of saprotrophic fungi tend to correspond with lower SOC content [84].

### 4.5. Effects of Soil Properties on Microbial Communities

Soil microbial communities in forest environments are regulated by multiple environmental variables [99]. Previous studies have shown that soil pH and nutrient levels can influence the structure and diversity of microbial communities [100,101]. In this study, pH was the dominant factor in explaining bacterial and fungal variation (Figure 8, Table S7), a result supported by previous studies [99,102,103]. Soil pH may directly or indirectly affect microbial diversity by changing soil organic matter (SOM) or nitrogen availability. At the same time, pH may change the interactions between bacteria; when conditions become more acidic, rhizospheric bacteria often engage less in competitive behaviors, potentially resulting in shifts in their abundance and composition. This study also supports that Actinobacteria become more abundant as soil pH rises [104,105]. The study also found that total nitrogen and total potassium in the soil make a certain contribution to the composition of bacterial communities (Figure 8a, Table S7). In the study, *Sphingomonas* was positively associated with soil total nitrogen (Figure 8c). Total nitrogen has been reported to influence the structure of nitrogen-fixing bacterial communities [106]. Earlier studies have regarded *Sphingomonas* as important non-symbiotic nitrogen-fixing (ANF) bacteria [76], which do not need to coexist with plants. Through biological nitrogen fixation, *Sphingomonas* contributes to improving soil fertility and enhancing nitrogen cycling processes in the soil ecosystem [107]. In addition, TK and AK are also key factors affecting the distribution of fungal flora (Figure 8b, Table S7) [29]. As an essential macronutrient for plant development, potassium availability can be influenced by bacterial activity, which alters its solubility and accessibility in soil, subsequently shaping plant growth and the recruitment of potassium-associated microbial taxa. The relative abundance of *Humicola* was significantly negatively correlated with N:P, TK, and AK (Figure 8d). *Humicola* is involved in the transformation processes of soil carbon and nitrogen, including denitrification [108], which can produce a variety of lignocellulose-degrading enzymes, increase soil soluble carbon sources, promote microbial metabolic activities, and thus affect soil nutrient content.

## 5. Conclusions

Our results demonstrate that soil nutrients were more enriched in mixed forests than in *Pinus tabulaeformis* and *Quercus acutissima* forests, as indicated by the significantly higher content of total potassium, available potassium, and organic carbon. Soil fungal alpha diversity was markedly influenced by both forest type and soil depth, in which the fungal diversity index of *Pinus tabulaeformis* was significantly the highest, and decreased with the soil depth. Mixed forests exhibited more complex microbial co-occurrence networks than pure forests, and the bacterial functions were dominated by chemoheterotrophy related to the carbon cycle and nitrification associated with the nitrogen cycle, and soil pH was found to be a key factor positively influencing most microbial functions. Symbiotrophs were the predominant group within the fungal community, with ectomycorrhizal fungi showing the greatest relative abundance in mixed forest ecosystems; the saprotrophic type mainly consisted of plant saprotrophs and wood saprotrophs, and the *Pinus tabulaeformis* forest exhibited a notably greater proportion compared to that observed in the mixed forests. RDA and correlation analysis showed that pH, TN, and AK were the key factors influencing the structure of bacterial communities. On the other hand, pH, N:P, TK, and AK were important variables affecting the distribution of fungi. In conclusion, fungal communities responded more sensitively to forest type, and mixed forests significantly enhanced soil fertility and fostered more complex microbial co-occurrence networks, thereby enhancing the stability and functional diversity of the soil microecosystem.

Current studies based on a single time point may underestimate the seasonal dynamics of soil microbial communities. It is recommended that future studies incorporate temporal sampling to better elucidate the spatiotemporal dynamics of microbial communities under near-natural forest management. Moreover, as this study focused on only three forest types, further research is needed to explore how forest type diversification influences soil microbial ecological processes.

## Figures and Tables

**Table 1:** Basic information of the sample site.

Type	Forest Age/a	Slope/°	Altitude/m	Density/ (Trees·hm^2^)	Average Breast Diameter/cm	Average Tree Height/m
QA	34	15	950	1095	15.3	8.9
PT	43	15	980	900	20.1	8.8
MF	42	18	1000	1100	14.8	6.2

**Table 2:** Permutational multivariate analysis of variance (PERMANOVA) of soil bacterial and fungal communities based on ASV levels.

Taxa	Factor	Df	Sum of Sqs	R^2^	F	Pr (>F)	Sig.
Bacteria	Type	2	0.2421	0.2602	3.7240	0.001	***
	Depth	2	0.0283	0.0305	0.4359	0.981	
	Depth/Type	4	0.0748	0.0804	0.5749	0.975	
	Residual	18	0.5852	0.6289			
	Total	26	0.9304	1.0000			
Fungi	Type	2	3.7608	0.2354	3.5807	0.024	*
	Depth	2	0.7881	0.0493	0.7503	0.552	
	Depth/Type	4	1.9741	0.1236	0.9398	0.491	
	Residual	18	9.4526	0.5917			
	Total	26	15.9756	1.0000			

Note: ***—*p* < 0.001; *—*p* < 0.05.

**Table 3:** Microbial co-occurrence network parameters for the three forest types.

Microbial Taxonomy	Network	Nodes/ Edges	Positive Edges/Negative Edges	Average Degree	Average Path Length	Density	Clustering Coefficient	Modularity	Complexity Index
Bacterial	QA	392/4790	84.05/15.95	24.429	2.834	0.063	0.653	0.602	0.034
	PT	395/3382	69.34/30.66	17.124	2.881	0.043	0.574	0.635	−0.922
	MF	407/5745	86.77/13.23	28.231	2.640	0.070	0.642	0.547	0.888
Fungi	QA	130/477	90.78/9.22	7.338	3.203	0.057	0.681	0.723	−0.342
	PT	166/695	74.68/25.32	8.373	3.186	0.051	0.525	0.598	−0.089
	MF	93/520	86.54/13.46	11.183	2.564	0.122	0.676	0.520	0.431

## Data Availability

The original contributions presented in this study are included in the article/Supplementary Materials. Further inquiries can be directed to the corresponding author.

## Supplementary Materials

The following supporting information can be downloaded at https://www.mdpi.com/article/10.3390/microorganisms13081906/s1. Table S1. Two-way ANOVA for soil physicochemical properties; Table S2. Two-way ANOVA for soil microbial diversity index; Table S3. Two-way ANOVA of bacteria with relative abundance > 1% at phylum and genus levels; Table S4. Two-way ANOVA of fungi with relative abundance > 1% at phylum and genus levels; Table S5. Two-way ANOVA of bacterial function with relative abundance > 1%; Table S6. Two-way ANOVA of fungal function; Table S7. Explanation and significance of environmental factors in bacterial and fungal community structure.
