# Supplementary material for: Effects of Near-Natural Forest Management on Soil Microbial Communities in the Temperate–Subtropical Transition Zone of China
Source: Microorganisms. 2025 Aug 15;13(8):1906. doi: 10.3390/microorganisms13081906 (PMC12388296; doi:10.3390/microorganisms13081906)
Supplement: Supplementary file 1 [file microorganisms-13-01906-s001.zip › microorganisms-3779857-supplementary.pdf]

Table S1 Two-way ANOVA for soil physicochemical properties

Variable	Effect	df	sum_sq	F	Pr(>F)	Sig.
BD	Forest	2	0.0663	1.4961	0.2506	
	Depth	2	0.1915	4.319	0.0294	*
	Forest:Depth	4	0.3493	3.94	0.0181	*
	Residual	18	0.3991			
PO	Forest	2	41.4550	0.3273	0.7251	
	Depth	2	814.7489	6.4326	0.0078	**
	Forest:Depth	4	1657.8225	6.5444	0.0020	**
	Residual	18	1139.9339			
pH	Forest	2	0.5724	6.4055	0.0079	**
	Depth	2	0.0100	0.1120	0.8947	
	Forest:Depth	4	0.0352	0.1969	0.9368	
	Residual	18	0.8042			
TP	Forest	2	0.0177	2.9940	0.0754	
	Depth	2	0.1524	25.8455	0.0000	***
	Forest:Depth	4	0.0014	0.1179	0.9744	
	Residual	18	0.0531			
TK	Forest	2	410.3318	21.1948	0.0000	***
	Depth	2	56.2279	2.9043	0.0807	
	Forest:Depth	4	18.5049	0.4779	0.7515	
	Residual	18	174.2401			
TN	Forest	2	1.1726	9.7366	0.0014	**
	Depth	2	5.4571	45.3129	0.0000	***
	Forest:Depth	4	1.1367	4.7192	0.0088	**
	Residual	18	1.0839			
AK	Forest	2	7175.6752	10.6959	0.0009	***
	Depth	2	5903.4508	8.7995	0.0022	**
	Forest:Depth	4	333.1542	0.2483	0.9069	
	Residual	18	6037.9327			
SOM	Forest	2	288.0878	10.1174	0.0011	**
	Depth	2	4978.4048	174.8373	0.0000	***
	Forest:Depth	4	460.1351	8.0798	0.0007	***
	Residual	18	256.2705			
SOC	Forest	2	155.8188	10.1973	0.0011	**
	Depth	2	1856.6370	121.5047	0.0000	***
	Forest:Depth	4	116.9188	3.8258	0.0202	*
	Residual	18	137.5233			
C:N	Forest	2	0.0385	2.8023	0.0872	

	Depth	2	1.5188	110.5928	0.0000	***
	Forest:Depth	4	0.0637	2.3207	0.0962	
	Residual	18	0.1236			
C:P	Forest	2	4.9646	14.5897	0.0002	***
	Depth	2	47.5907	139.8572	0.0000	***
	Forest:Depth	4	3.5815	5.2625	0.0055	**
	Residual	18	3.0625			
N:P	Forest	2	14.1170	29.1040	0.0000	***
	Depth	2	1.4581	3.0062	0.0747	
	Forest:Depth	4	0.5015	0.5169	0.7243	
	Residual	18	4.3655			

Note:***:$P < 0.001$; **: $P < 0.01$; *:$P < 0.05$

Table S2 Two-way ANOVA for soil microbial diversity index

Type	Index	Effect	Df	Mean Sq	F value	Pr(>F)	Sig.
Bacterial	OTU	Forest	2	129890.482	0.830	0.455	
		Depth	2	575593.815	3.647	0.047	*
		Forest:Depth	4	122167.482	0.774	0.556	
	Shannon	Forest	2	0.097	0.956	0.403	
		Depth	2	0.162	1.593	0.231	
		Forest:Depth	4	0.0406	0.400	0.806	
	Chao1	Forest	2	161376.432	0.774	0.476	
		Depth	2	699931.9108	3.357	0.058	
		Forest:Depth	4	164905.1944	0.791	0.546	
Funfi	OTU	Forest	2	327134.926	80.279	0.000	***
		Depth	2	178024.482	43.687	0.000	***
		Forest:Depth	4	12454.037	3.056	0.044	*
	Shannon	Forest	2	2.706	6.110	0.009	**
		Depth	2	2.186	4.936	0.020	*
		Forest:Depth	4	0.3483	0.787	0.549	
	Chao1	Forest	2	329214.299	55.037	0.000	***
		Depth	2	184430.060	30.832	0.000	***
		Forest:Depth	4	14352.112	2.399	0.088	

Note:***:$P<0.001$; **: $P<0.01$; *:$P<0.05$

Table S3 Two-way ANOVA of bacteria with relative abundance > 1 % at phylum and genus levels

	Taxa	Source	Df	Mean Sq	F value	Pr(>F)	Sig.
phylum	Proteobacteria	Forest	2	0.0196	3.5216	0.0512	
		Depth	2	0.0114	2.0518	0.1575	

		Forest:Depth	4	0.0095	1.7078	0.1921	
	Actinobacteriota	Forest	2	0.0159	5.2034	0.0165	*
		Depth	2	0.0002	0.0599	0.9421	
		Forest:Depth	4	0.0005	0.1558	0.9578	
	Acidobacteriota	Forest	2	0.0015	0.3933	0.6805	
		Depth	2	0.0005	0.1331	0.8763	
		Forest:Depth	4	0.0045	1.165	0.359	
	Gemmatimonadota	Forest	2	0.0009	2.243	0.135	
		Depth	2	0.0001	0.2991	0.7451	
		Forest:Depth	4	0.0001	0.2765	0.8893	
	Chloroflexi	Forest	2	0.0028	6.2459	0.0087	**
		Depth	2	0.0008	1.8611	0.1842	
		Forest:Depth	4	0.0006	1.3157	0.3019	
	Bacteroidota	Forest	2	0.0006	4.1427	0.0331	*
		Depth	2	0.0002	1.1177	0.3487	
		Forest:Depth	4	0.0001	0.3595	0.8341	
	Myxococcota	Forest	2	0.0001	0.9792	0.3948	
		Depth	2	0.0002	2.64	0.0988	
		Forest:Depth	4	0.0001	1.0977	0.3877	
	Methylomirabilota	Forest	2	0.0057	4.5296	0.0255	*
		Depth	2	0.0017	1.3156	0.2929	
		Forest:Depth	4	0.001	0.8333	0.5215	
	Verrucomicrobiota	Forest	2	0.0001	0.1953	0.8243	
		Depth	2	0.0002	0.6664	0.5258	
		Forest:Depth	4	0.0003	0.7977	0.5422	
	Latescibacterota	Forest	2	0.0005	4.0176	0.0361	*
		Depth	2	0.0002	1.8685	0.1831	
		Forest:Depth	4	0.0001	0.6607	0.6273	
	RB41	Forest	2	7.5137	18.2124	<0.001	***
		Depth	2	2.2016	5.3364	0.0151	*
		Forest:Depth	4	3.5822	8.6828	0.0004	***
	Sphingomonas	Forest	2	1.2030	5.0399	0.0183	*
		Depth	2	0.3953	1.6562	0.2187	
		Forest:Depth	4	0.3786	1.5861	0.2210	
	Gemmatimonas	Forest	2	4.1100	2.8393	0.0848	
		Depth	2	0.7214	0.4984	0.6157	
		Forest:Depth	4	2.8699	1.9826	0.1404	
genus	*Haliangium*	Forest	2	1.6458	3.9047	0.0390	*
		Depth	2	0.4925	1.1684	0.3333	
		Forest:Depth	4	0.3929	0.9321	0.4675	
	Candidatus_Solibacter	Forest	2	0.1079	4.1215	0.0336	*
		Depth	2	0.0217	0.8275	0.4531	
		Forest:Depth	4	0.0279	1.0666	0.4017	
	MND1	Forest	2	0.1382	2.7421	0.0913	

		Depth	2	0.0726	1.4413	0.2627	
		Forest:Depth	4	0.0369	0.7315	0.5822	
	Reyranella	Forest	2	0.0188	0.3625	0.7009	
		Depth	2	0.0309	0.5960	0.5615	
		Forest:Depth	4	0.0209	0.4035	0.8037	
	Candidatus_Udaeobacter	Forest	2	0.1458	1.9506	0.1711	
		Depth	2	0.0100	0.1335	0.8759	
		Forest:Depth	4	0.0519	0.6945	0.6055	
	Bryobacter	Forest	2	9.3734	8.1635	0.0030	**
		Depth	2	1.9106	1.6640	0.2172	
		Forest:Depth	4	1.6554	1.4417	0.2610	
	Dongia	Forest	2	0.1498	4.1759	0.0324	*
		Depth	2	0.0436	1.2158	0.3197	
		Forest:Depth	4	0.1023	2.8527	0.0541	
	Bradyrhizobium	Forest	2	2.0899	0.8061	0.4621	
		Depth	2	2.9090	1.1221	0.3473	
		Forest:Depth	4	1.5062	0.5810	0.6803	
	Ellin6067	Forest	2	1.6809	5.1272	0.0173	*
		Depth	2	0.1011	0.3083	0.7385	
		Forest:Depth	4	0.2327	0.7099	0.5957	

Note:***:*P*<0.001; **: *P*<0.01; *:*P*<0.05

Table S4 Two-way ANOVA of fungi with relative abundance > 1 % at phylum and genus levels

	Taxa	Source	Df	Mean Sq	F value	Pr(>F)	Sig.
	Basidiomycota	Forest	2	0.1775	3.3041	0.0599	
		Depth	2	0.0523	0.9733	0.3969	
		Forest:Depth	4	0.0261	0.4851	0.7465	
	Ascomycota	Forest	2	0.0467	3.8497	0.0406	*
		Depth	2	0.0308	2.5392	0.1068	
		Forest:Depth	4	0.0208	1.7120	0.1912	
	Mortierellomycota	Forest	2	0.0059	4.6975	0.0228	*
phylum		Depth	2	0.0002	0.1295	0.8793	
		Forest:Depth	4	0.0014	1.0933	0.3896	
	Rozellomycota	Forest	2	0.0018	0.8183	0.4569	
		Depth	2	0.0024	1.0790	0.3609	
		Forest:Depth	4	0.0022	0.9662	0.4500	
	Chytridiomycota	Forest	2	0.0004	1.3591	0.2820	
		Depth	2	0.0004	1.1196	0.3481	
		Forest:Depth	4	0.0003	0.8961	0.4866	
	Aspergillus	Forest	2	1.4020	0.2053	0.8163	
		Depth	2	18.6221	2.7268	0.0924	
genus		Forest:Depth	4	7.4573	1.0920	0.3903	
	Humicola	Forest	2	0.5189	1.0598	0.3672	
		Depth	2	1.1940	2.4386	0.1156	

	Forest:Depth	4	0.3396	0.6937	0.6060	
Hymenogaster	Forest	2	126.0300	1.4848	0.2530	
	Depth	2	8.5727	0.1010	0.9044	
	Forest:Depth	4	55.2292	0.6507	0.6338	
Ilyonectria	Forest	2	1.4223	9.4339	0.0016	**
	Depth	2	0.1644	1.0902	0.3574	
	Forest:Depth	4	0.2255	1.4959	0.2452	
Inocybe	Forest	2	211.8696	1.5161	0.2463	
	Depth	2	3.4688	0.0248	0.9755	
	Forest:Depth	4	96.1706	0.6882	0.6095	
Membranomyces	Forest	2	1551.9069	4.4497	0.0269	*
	Depth	2	41.2198	0.1182	0.8892	
	Forest:Depth	4	40.9960	0.1175	0.9745	
Mortierella	Forest	2	25.9267	19.2218	0.0000	***
	Depth	2	4.3278	3.2086	0.0643	
	Forest:Depth	4	2.9396	2.1794	0.1126	
Peziza	Forest	2	9.7191	1.0069	0.3850	
	Depth	2	8.4548	0.8760	0.4335	
	Forest:Depth	4	6.7137	0.6956	0.6048	
Russula	Forest	2	716.4751	2.1035	0.1510	
	Depth	2	152.4691	0.4476	0.6461	
	Forest:Depth	4	87.1920	0.2560	0.9022	
Sebacina	Forest	2	1961.5568	10.0828	0.0012	**
	Depth	2	431.5880	2.2185	0.1377	
	Forest:Depth	4	183.7163	0.9443	0.4611	
Thelephora	Forest	2	736.2499	2.7338	0.0919	
	Depth	2	5.3545	0.0199	0.9803	
	Forest:Depth	4	11.0564	0.0411	0.9965	
Tomentella	Forest	2	4.8842	2.8763	0.0824	
	Depth	2	0.2574	0.1516	0.8604	
	Forest:Depth	4	0.2550	0.1502	0.9605	
Trichoderma	Forest	2	0.8932	2.2426	0.1350	
	Depth	2	0.8754	2.1979	0.1399	
	Forest:Depth	4	0.5258	1.3201	0.3003	

Note:***:P<0.001; **: P<0.01; *:P<0.05

Table S5 Two-way ANOVA of bacterial function with relative abundance > 1 %

Function	Source	sum_sq	df	F	PR(>F)	Sig.
aerobic_ammonia_oxidation	Forest	8.4690	2	1.1018	0.3537	
	Depth	3.0510	2	0.3969	0.6781	
	Forest:Depth	5.7905	4	0.3767	0.8223	
	Residual	69.1808	18			
aerobic_chemoheterotrophy	Forest	140.3528	2	10.4762	0.0010	**
	Depth	82.2493	2	6.1393	0.0093	**

	Forest:Depth	12.7068	4	0.4742	0.7541	
	Residual	120.5752	18			
chemoheterotrophy	Forest	34.1377	2	1.4217	0.2671	
	Depth	51.9469	2	2.1635	0.1439	
	Forest:Depth	23.2623	4	0.4844	0.7470	
	Residual	216.1001	18			
nitrate_reduction	Forest	0.8886	2	1.5892	0.2314	
	Depth	0.1279	2	0.2288	0.7978	
	Forest:Depth	0.6454	4	0.5771	0.6829	
	Residual	5.0324	18			
nitrification	Forest	8.4724	2	1.1026	0.3534	
	Depth	3.0499	2	0.3969	0.6781	
	Forest:Depth	5.7790	4	0.3760	0.8227	
	Residual	69.1572	18			
nitrogen_fixation	Forest	3.2600	2	2.7142	0.0933	
	Depth	4.0473	2	3.3697	0.0571	
	Forest:Depth	0.8390	4	0.3493	0.8411	
	Residual	10.8098	18			
predatory_or_exoparasitic	Forest	0.1570	2	0.9282	0.4134	
	Depth	0.2970	2	1.7555	0.2011	
	Forest:Depth	0.3807	4	1.1251	0.3758	
	Residual	1.5227	18			
ureolysis	Forest	3.1564	2	2.0053	0.1636	
	Depth	2.0238	2	1.2857	0.3007	
	Forest:Depth	4.3883	4	1.3939	0.2758	
	Residual	14.1667	18			

Table S6 Two-way ANOVA of fungal function

Function	Source	sum_sq	df	F	PR(>F)	Sig.
Ectomycorrhizal	Forest	4850.0961	2	5.6287	0.0126	*
	Depth	15.6913	2	0.0182	0.9820	
	Forest:Depth	314.7460	4	0.1826	0.9444	
	Residual	7755.0467	18			
Endophyte	Forest	46.5067	2	2.9821	0.0761	
	Depth	6.8262	2	0.4377	0.6522	
	Forest:Depth	17.7740	4	0.5698	0.6879	
	Residual	140.3598	18			
Root Associated Biotroph	Forest	636.6588	2	8.7463	0.0022	**
	Depth	30.8811	2	0.4242	0.6606	
	Forest:Depth	82.9163	4	0.5695	0.6881	
	Residual	655.1243	18			
Orchid Mycorrhizal	Forest	634.9533	2	8.7590	0.0022	**
	Depth	30.4770	2	0.4204	0.6631	
	Forest:Depth	82.7327	4	0.5706	0.6873	
	Residual	652.4229	18			

Plant Saprotroph	Forest	48.0238	2	3.8048	0.0419	*
	Depth	0.3864	2	0.0306	0.9699	
	Forest:Depth	21.4245	4	0.8487	0.5128	
	Residual	113.5970	18			
Wood Saprotroph	Forest	3.8074	2	0.3623	0.7010	
	Depth	2.4006	2	0.2284	0.7981	
	Forest:Depth	10.5444	4	0.5017	0.7349	
	Residual	94.5825	18			
Animal Pathogen	Forest	2.8124	2	0.3202	0.7301	
	Depth	5.9429	2	0.6766	0.5208	
	Forest:Depth	2.1611	4	0.1230	0.9724	
	Residual	79.0494	18			
Fungal Parasite	Forest	14.7677	2	2.4251	0.1168	
	Depth	13.5427	2	2.2240	0.1370	
	Forest:Depth	20.6365	4	1.6945	0.1951	
	Residual	54.8048	18			
Plant Pathogen	Forest	11.6101	2	1.3051	0.2956	
	Depth	3.3585	2	0.3775	0.6909	
	Forest:Depth	17.7946	4	1.0001	0.4331	
	Residual	80.0641	18			

Table S7 Explanation and significance of environmental factors on bacterial and fungal community structure

Variable	Bacterial		Fungi	
	R^2	P	R^2	P
BD	0.0745	0.398	0.0277	0.737
PO	0.0413	0.607	0.0072	0.911
PH	0.3483	0.011	0.7524	0.001
TP	0.1395	0.140	0.1066	0.257
TK	0.1449	0.164	0.5082	0.002
TN	0.2768	0.015	0.1647	0.113
AK	0.2238	0.044	0.2398	0.031
SOM	0.0307	0.697	0.0031	0.970
SOC	0.0363	0.641	0.0186	0.805
C:N	0.0530	0.516	0.0001	0.999
C:P	0.0485	0.542	0.0293	0.702
N:P	0.1702	0.118	0.5940	0.001
